# Clinical characteristics and risk factors of *Clostridioides difficile* infection in children with diarrhoea

**DOI:** 10.3389/fped.2025.1430803

**Published:** 2025-02-12

**Authors:** Ning Wang, Kun Jiang, Jinyue Huang, Mengzhu Hou, Lu Wang, Wei Wang, Yulian Fang

**Affiliations:** ^1^ Tianjin Pediatric Research Institute, Tianjin Key Laboratory of Birth Defects for Prevention and Treatment, Tianjin Children’s Hospital (Children’s Hospital of Tianjin University), Tianjin, China; ^2^ Department of Respiratory, Tianjin Children’s Hospital (Children’s Hospital of Tianjin University), Tianjin, China

**Keywords:** *Clostridioides difficile*, children, diarrhea, risk factor, clinical characteristic

## Abstract

**Objective:**

To investigate the clinical characteristics and risk factors of *Clostridioides difficile* infection (CDI) in children with diarrhea, and to provide evidence for the clinical prevention and treatment of CDI.

**Methods:**

The clinical data of 192 children with diarrhoea suspected of CDI admitted from August 2020 to March 2023 were retrospectively analysed and divided into two groups according to whether CDI occurred, and the clinical characteristics and risk factors of the two groups were analysed statistically.

**Results:**

There were 85 cases of CDI in children with diarrhoea, 60 males (70.6%) and 25 females (29.4%), with a male to female ratio of 2.4:1; clinical manifestations were mostly fever, diarrhoea, abdominal pain, vomiting and blood in stool, with no statistically significant differences compared to the non-infected group. Interleukin - 6 (IL - 6) levels were lower in the CDI group than in the non - CDI group, and the difference was statistically significant (*P* < 0.05). Multi-factor logistic regression analysis was used to show that a history of hospitalisation within the last three months, a history of antibiotic treatment for ≥7 days, non-breastfeeding, and a history of antibiotic combined with probiotic treatment were risk factors for CDI in children with diarrhoea (*P* < 0.05).

**Conclusions:**

A history of hospitalisation within the last three months, previous antibiotic treatment for ≥7 days or combined with probiotic treatment, and non-breastfeeding are risk factors for CDI in children with diarrhoea, so children with diarrhoea who present as described above need to be alerted to CDI and are advised to have active investigations to facilitate rapid and effective control of the disease and improve prognosis.

## Introduction

*Clostridioides difficile* (CD) is a specialized anaerobic, grossly gram-positive bacterium transmitted mainly by the faecal-oral route and is an important cause of nosocomial infections associated with gastroenteritis (1, 2). *Clostridioides difficile* infection (CDI) mainly cause diarrhoeal discomfort in patients and in severe cases can lead to pseudomembranous enterocolitis, toxic megacolon, intestinal necrosis and even life-threatening (3, 4). From the 1990s to the present, countries have reported increasing incidence of CDI and a rising trend in disease severity and mortality rates, The mortality rate of CDI patients is about 6.9%–16.7%, making it a serious public health problem (5). Since 2000, the incidence rate of CDI has been rising, especially the outbreak of high virulence RT027/NAP1/BI in Europe and the United States, and the case fatality rate has also gradually increased. At present, strain 027 (A + B + CDT+) has appeared in all provinces of Canada and at least 40 states of the United States, which has become a health problem affecting global public health. Before 2010, there were few data related to CD research in Asia. Since 2013, more and more research results show that the incidence rate of CDI in Asia is also rising rapidly (6). The incidence rate of CDI in Asia (53/10,000) is close to the average incidence rate of EUCLID study in Europe (70/10,000) and the average incidence rate of hospitalized patients in the United States (54/10,000) (7, 8). Toxins are the main pathogenic factor of CDI, and toxin A is enterotoxin, which mainly causes intestinal inflammation and leads to intestinal wall bleeding and necrosis; Toxin B is a cytotoxic substance that can stimulate monocytes to release inflammatory cytokines, directly damaging intestinal wall cells and even causing pseudomembranous colitis in severe cases. The CD strains prevalent in European and American countries are mainly tcdA^+^tcdB^+^, but the 027 strain is a sporadic case in Asia. The ST37 strain, which is negative for toxin A and positive for toxin B (A^−^B^+^), is more prevalent in Asia (9, 10). CDI is mainly caused by the severe imbalance of gut microbiota due to the overuse of antibiotics, leading to the proliferation of CD in the intestine. For example, the abuse of broad-spectrum antibiotics can inhibit the growth of CD, but at the same time, it can also inhibit the growth of normal microbiota, causing intestinal microbiota disorder and reducing CD colonization resistance, leading to the recurrence of CDI. However, the pathological and clinical manifestations of CDI in children and adults are not the same, and the gut microbiome of children is in a dynamic state of change (11, 12). Therefore, the direct application of adult clinical medication regimens to children is controversial. Furthermore, due to the insufficient diagnostic capabilities and expensive testing costs of *Clostridioides difficile* laboratories in China, the testing of *Clostridioides difficile* cannot be carried out routinely in clinical practice. Therefore, analyzing the high-risk factors for CDI in diarrhea patients and establishing a predictive model to screen for CDI is of great significance for reducing the missed detection rate of CDI and improving the accuracy of treatment. In this study, the clinical characteristics of CDI in children with diarrhoea and its susceptibility factors were investigated to provide a scientific basis and theoretical foundation for the risk assessment of CDI in children with diarrhoea, and to provide clues for early clinical prevention and treatment to facilitate rapid and effective control of the disease and improve prognosis.

## Methods

A total of 192 children who met the inclusion criteria were selected from 527 children with suspected CDI admitted to the internal medicine department of Tianjin Children's Hospital (Children's Hospital of Tianjin University) from August 2020 to March 2023. The study procedure was approved by the ethics committee of the Tianjin Children's Hospital (2024-SBKT-046). Written informed consent was obtained from parent or guardian of all participants. Inclusion criteria: (1) age <18 years old; (2) according to the Clinical Practice Guidelines for CDI in Adults and Children released by the American Society of Infectious Diseases and the American Society of Healthcare Epidemiology in 2017 (10), CDI diagnosis has corresponding clinical symptoms such as fever, abdominal pain, diarrhea, and positive for any of the following indicators: (1) toxic CD detected in feces; (2) positive CD toxin detection in feces; (3) colonoscopy or histopathological examination shows pseudomembranous colitis (10); (3) initial diagnosis of CDI; (4) clinical and laboratory data are complete. Exclusion criteria: Other infectious and noninfectious causes of diarrhea were excluded for all patients, where the presence of potential pathogens for children younger than 2 years of age was tested using the multiplex polymerase chain reaction gastrointestinal pathogens panel. Excluded cases with incomplete clinical data (incomplete clinical data included cases that were not recorded in the complete treatment record or dropped out of the study or were discharged automatically and could not be followed up).

### Clinical grouping

The positive cases of *Clostridioides difficile* toxin A/B(CDAB) and glutamate dehydrogenase (GDH) detected by ELISA were divided into infection group, and the negative cases were divided into non-infection group. The Cepheid GeneXpert instrument and the accompanying *Clostridioides difficile* test kit (GeneXpert CD) were used for *Clostridioides difficile* genetic testing, and those with positive test results were divided into infected groups.

General information (gender, age, etc.), symptoms on admission (fever, diarrhoea, vomiting, etc.), medication history prior to admission (antibiotics, probiotics, etc.), laboratory results within 48 h of admission (white blood cell count, neutrophil percentage, C-reactive protein, calcitoninogen, etc.), radiological examinations (x-rays or CT, ultrasound), etc. were collected from children who met the inclusion criteria through our electronic medical record system.

All statistical analyses were performed using SPSS 25.0. The measurement data conforming to the normal distribution were expressed by mean ± standard deviation and the two-sample *T* test was used for inter-group comparison. Measurement data with non-normal distribution were represented by median (M) and interquartile range (P_25_, P_75_), and Mann–Whitney *U* test was used for comparison between groups. Counting data were expressed as frequency and percentage (%), and *χ*^2^ test was used for comparison between groups. The risk factors were analyzed by multifactor regression. A *P*-value of less than 0.05 was considered statistically significant.

## Results

Among 527 patients with suspected CDI, 192 children with diarrhea meeting the criteria were included. Among them, 85 cases (44.3%) were positive for CDI and 107 cases (55.7%) were negative. There was no statistically significant difference between the two groups compared to gender, age and clinical symptoms such as fever, bloating, abdominal pain, vomiting and blood in stool (*p* > 0.05), Table 1.

**Table 1:** Comparison of clinical features between the *C. difficile* group and the No *C. difficile* group.

Variables	*C. difficile* (*n* = 85)	No *C. difficile* (*n* = 107)	*Χ*^2^/*Z*	*P*
Gender (Male/Female)	60/25	63/44	2.821	0.093
Age [M (P_25_, P_75_), m]	12.0 (10.00, 42.00)	24.00 (8.00,108.00)	−1.337	0.181
Hospital stay [M (P_25_, P_75_), d]	7.00 (5.00, 9.5)	6.00 (4.00, 8.00)	−1.587	0.112
Symptoms				
Fever	32 (37.6%)	51 (47.7%)	1.937	0.164
Bloating	5 (5.9%)	5 (4.7%)	0.002	0.962
Abdominal pain	24 (28.2%)	43 (40.1%)	2.978	0.084
Vomit	11 (12.9%)	25 (23.4%)	3.378	0.066
Hematochezia	55 (64.7%)	58 (54.2%)	2.157	0.142

m, months; d, days.

The differences in IL-6 compared between the two groups were statistically significant (*p* < 0.05), while the differences in other laboratory indicators were not statistically significant (*p* > 0.05) Table 2.

**Table 2:** Comparison of peripheral blood tests between the *C. difficile* group and the No *C. difficile* group.

Laboratory index	*C. difficile* (*n* = 85)	No *C. difficile* (*n* = 107)	*Χ*^2^/*Z*	*P*
WBC (4.6–11.9 × 10^9^/L)	9.51 (6.08,12.11)	8.87 (5.87,12.11)	−0.107	0.915
NEUT (32%–71%)	37.96 (26.85,52.42)	42.5 (27.0,55.60)	−1.394	0.163
RBC (4.30–5.70 × 10^12^/L)	4.49 ± 0.38	4.53 ± 0.46	−0.541	0.589
Hb (121–158 g/L)	120.26 ± 11.62	123.03 ± 15.90	−1.389	0.166
PLT (177–446 × 10^9^/L)	325.11 (255.35,325.00)	334.00 (237.86,407.00)	−0.348	0.728
GLU (3.90–6.10 mmol/L)	5.04 (4.42,5.76)	5.23 (4.56,5.92)	−1.141	0.254
TP (65.0–84.0 g/L)	63.70 (59.60,67.65)	65.20 (60.40,69.00)	−0.747	0.455
Alb (39.0–54.0 g/L)	44.60 (42.55,46.65)	43.23 (41.10,45.80)	−1.881	0.060
AST (14.0–44.0 U/L)	39.00 (26.00,51.00)	36.00 (25.00,45.00)	−1.636	0.102
CK-MB (0–24.0 U/L)	12.47 (7.00,21.50)	13.00 (6.00,20.00)	−0.504	0.615
La (0.50–2.20 mmol/L)	3.04 (2.36,3.69)	2.71 (2.03,3.49)	−1.726	0.084
SCr (27.0–66.0 umol/L)	25.00 (19.00,31.00)	26.00 (21.00,39.00)	−1.856	0.063
CRP (0–8.0 mg/L)	2.50 (2.50,7.20)	3.53 (2.50,11.49)	−1.852	0.064
PCT (0–0.05 ng/ml)	0.07 (0.04,0.13)	0.08 (0.04,0.19)	−1.518	0.129
IL-6 (0–7 pg/ml)	2.10 (1.50,9.21)	4.14 (1.54,13.84)	−2.583	0.010

WBC, white blood cells; NEUT%, neutrophil ratio; RBC, red blood cells; Hb, hemoglobin; PLT, platelet count; GLU, glucose; TP, total protein; Alb, albumin; AST, aspartate aminotransferase; CK-MB, creatine kinase MB isoenzyme; LA, blood lactate; SCr, serum creatinine; CRP, C-reactive protein; PCT, procalcitonin; IL-6, interleukin-6.

The differences between the two groups were statistically significant (*P* < 0.05) for history of hospitalisation within the last 3 months, non-breastfeeding, history of antibiotic treatment for ≥7 days, and history of antibiotic combined with probiotic treatment (Table 3).

**Table 3:** Comparison of risk factors between the *C. difficile* group and the No *C. difficile* group.

Variables	*C. difficile* (*n* = 85)	No *C. difficile* (*n* = 107)	*T*/*Z*	*P*
With underlying disease	16 (18.8%)	19 (17.8%)	0.036	0.849
History of hospitalisation within the last 3 months	14 (16.5%)	7 (6.5%)	4.794	0.029
History of premature birth	10 (11.8%)	8 (7.5%)	1.025	0.311
Non-breastfeeding	37 (43.5%)	29 (27.1%)	5.666	0.017
History of antibiotic exposure	52 (61.2%)	53 (49.5%)	2.592	0.107
History of antibiotic treatment for ≥7 days	18 (21.2%)	10 (9.3%)	5.323	0.021
History of probiotic exposure	26 (30.6%)	23 (21.5%)	2.061	0.151
History of probiotic treatment ≥7 days	5 (5.9%)	5 (4.7%)	0.002	0.962
History of antibiotic combined with probiotic treatment	20 (23.5%)	9 (8.4%)	8.443	0.004

The factors that were statistically different in the univariate analysis were used as independent variables and the presence or absence of CDI was used as a response variable, and the results of the multifactorial logistic regression analysis showed History of hospitalisation within the last three months, history of antibiotic treatment for ≥7 days, non-breastfeeding, and history of antibiotic combined with probiotic therapy were independent risk factors for CDI (*P* < 0.05) (Table 4). For specific records of antibiotic use, see Tables 5 and 6.

**Table 4:** Multi-factor logistic regression analysis of *clostridioides difficile* infection in children with diarrhoea.

Variables	*B*	SE	Wald	*P*	OR	95% CI
History of hospitalisation within the last 3 months	1.122	0.504	4.953	0.026	3.070	1.143–8.245
History of antibiotic treatment for ≥7 days	0.917	0.457	4.024	0.045	2.502	1.021–6.131
Non-breastfeeding	0.801	0.328	5.967	0.015	2.228	1.172–4.238
History of antibiotic combined with probiotic treatment	1.054	0.451	5.463	0.019	2.868	1.185–6.939

**Table 5:** Antibiotic use in patients treated with antibiotics combined with probiotics.

Types of antibiotics used	*C. Difficile* (*n* = 20)	No *C. difficile* (*n* = 9)
Cephalosporin-type antibiotics (specific names unknown)	6	—
Cefdinir	7	5
Cefixime	2	4
Cefdinir and cefixime	3	—
Cefotaxime sodium	1	—
Cefdinir and amoxicillin	1	—

**Table 6:** Antibiotic exposure in each group.

Types of antibiotics used	*C. Difficile* (*n* = 52)	No *C. difficile* (*n* = 53)
Antibiotics (specific names unknown)	2	1
Cephalosporin antibiotics (specific names unknown)	16	8
Only cefdinir	13	20
Cefdinir and amoxicillin	2	2
Cefdinir and erythromycin	1	—
Cefdinir and cefixime	3	1
Cefdinir and cefotaxime	—	1
Only cefixime	11	7
Cefoperazone sodium	1	—
Cefotaxime sodium	1	—
Ceftazidime	1	—
Fosfomycin sodium	1	—
Cefixime and amoxicillin	—	2
Cefixime and penicillin class antibiotics (specific names unknown)	—	1
Ceftriaxone	—	3
Penicillin class antibiotics (specific names unknown)	—	1
Amoxicillin	—	4
Azlocillin and penicillin G	—	1
Imipenem	—	1

## Discussion

Diarrhoeal diseases are a group of diseases characterised by increased stool frequency and altered stool characteristics, caused by a variety of pathogens and factors. Infectious diarrhoea is the most common cause of death in children <5 years of age in developing countries and is the 5th leading cause of death in the world, making it a major public health problem (13). CD is a conditionally pathogenic bacterium, mainly found in the intestinal tract and faeces of healthy humans and animals, accounting for less than 3% of the human intestinal flora, and the use of large amounts of antibacterial or acid-suppressive drugs can lead to disorders of the intestinal flora (14). With the misuse of broad-spectrum antimicrobial drugs and the emergence and spread of highly virulent strains of CD in recent years, the incidence of CDI has increased annually worldwide and is accompanied by increased health care costs and mortality, and is now recognised as the primary pathogen of nosocomially acquired enteric infections and antimicrobial drug-associated diarrhoea (2, 15, 16). Therefore, the understanding of clinical characteristics and risk factors of CDI in children in the hospital or community setting requires urgent clinical attention. Previous studies have suggested that the prevalence of CD in European and American countries is mainly tcdA^+^tcdB^+^ (8). This study shows that tcdA^−^tcdB^+^ is the main epidemiological feature of CDI in children, and the majority of strains with the tcdA^−^tcdB^+^ genotype are ST37, which is consistent with Jin's (17) report on the molecular epidemiology of CD in China.

It has been shown that approximately 15%–25% of antimicrobial drug-associated diarrhoea, 50%–75% of antimicrobial drug-associated colitis and 95%–100% of pseudomembranous enterocolitis are clinically caused by CDI (10, 18), which mainly presents with fever, abdominal pain, watery stool diarrhoea, vomiting and blood in the stool. No statistical differences were found in the present study in terms of symptomatic presentation, in agreement with a previous study by Borali et al. (19), suggesting that the clinical phenotype is not specific and cannot be easily differentiated from other pathogenic infections, and that follow-up laboratory tests need to be improved to clarify the diagnosis.

CDI is an infectious disease characterised by inflammatory lesions and the formation of pseudomembranes in the intestinal tract, caused by an overgrowth of toxin-producing CDs leading to the loss of intestinal flora and the release of toxins (18). It has been shown that a variety of inflammatory cytokines are involved in the development of CDI. Inflammatory cells such as neutrophils and macrophages can release inflammatory factors such as IL-6 and IFN-r, leading to pathological damage to the intestinal mucosa (20). The statistics in this study suggest that the levels of CRP and IL-6 in the CDI group were lower than those in the non-CDI group, contrary to the previous belief that CDI may cause higher inflammatory index coefficients. Analyzing the reasons, most of the children in the CDI group had received anti-infective treatment before admission, which may have suppressed the inflammatory response to a certain extent, resulting in a lower inflammation index in the CDI group than in the non-CDI group. However, due to the limitation of the sample size, it is necessary to further expand the sample size to verify this inference.

CDI is mostly associated with the use of broad-spectrum antibiotics, proton pump inhibitors, immunosuppressants and risk factors such as ageing, a long history of hospitalisation and underlying disease (21, 22). In this study, a history of previous hospitalisation within three months was found to be a risk factor for CDI, whereas underlying diseases were not. The reasons for this were: a history of previous hospitalisation within three months increases exposure to CD budding cells (23), and the frequency of repeated hospitalization was higher in children with CDI than in non-CDI children in the present study, which also suggests that environmental factors are prerequisites for CDI; and with regard to underlying diseases, there are differences in the types of underlying diseases between children and adults, as children tend to suffer from congenital diseases such as birth defects and metabolic disorders, which are mostly surgically treated or diet-controlled, whereas adults or older adults tend to have respiratory or cardiovascular disorders that require long-term medication, which may lead to disturbances in the intestinal flora and provide the underlying conditions for CDI.

A history of antibiotic exposure has been shown to be a risk factor for CDI in children (24–26), but this study found no statistically significant difference between the two groups, while a history of antibiotic treatment for ≥7 days was a high risk factor for CDI in children with diarrhoea. The reason for this is that prolonged antibiotic use can lead to a decrease in functional flora abundance and diversity, resulting in an increase in invasive flora and hence the development of CD (27). The clinical report by Goldenberg et al. (28) showed that the combination of probiotics and antibiotics reduced the risk of CDI by nearly 2.5%, which contradicts the finding of this study that the combination of antibiotics and probiotics was a risk factor for the development of CDI in children with diarrhoea. The main types of antibiotics used in the combination therapy group in this study are cefdinir and cefixime, which are third-generation cephalosporins and broad-spectrum antibiotics. Probiotics mainly include infant Bifidobacterium, Lactobacillus acidophilus, Enterococcus faecalis, and Bacillus cereus. The following reasons were considered: The case population included in this study was mainly children with diarrhea, while the study population of Goldenberg et al. (28) was mainly adults and elderly patients. The intestinal flora genera of the two populations were significantly different. The synergistic effect of the combination of broad-spectrum antibiotics and probiotics may more easily affect the intestinal microecological environment and lead to the destruction of the flora, which provides a basis for CDI. Moreover, studies have shown that the presence of antibiotics in the intestine can increase the production of intestinal endotoxins, the expression of germination and colonization factors (29, 30), which also provides a strong argument for the results of this study. However, the number of cases included in this study was limited, and CDI group accounted for a relatively high proportion of patients in antibiotic combined probiotic treatment, which may lead to bias in results. It is necessary to further expand the number of cases, specify the types of antibiotics, and conduct multi-center studies to support this conclusion.

This study also identified non-breastfeeding as a risk factor for CDI, which is presumably related to the fact that non-breastfeeding makes the child less resistant and tends to increase the burden on the gut, causing an imbalance in the physiological homeostasis of the gut and thus increasing the rate of infection. The study on the mechanism of intestinal disorders and CDI will be followed by the improvement of intestinal macro-genome sequencing analysis to further clarify the mechanism of intestinal disorders and CDI.

This study has some limitations: (i) it is a single-centre study with limited sample size and no healthy control group; (ii) this study is a retrospective analysis with no long-term follow-up results and there is a lack of follow-up information; (iii) admission bias (e.g., berkson bias) may arise when selecting cases among inpatients. Therefore, further multicentre and large sample studies will be conducted to analyse the risk factors for CDI in order to provide a theoretical basis for further clinical interventions.

## Conclusion

In summary, alterations in the intestinal microenvironment and combined organismal immune imbalances are central factors in the development and progression of CDI. A history of previous hospitalisation within 3 months, the use of antimicrobial drugs or combined probiotic therapy for longer than a certain period of time, and non-breastfeeding may increase the probability of CDI in children with diarrhoea. In such children with symptoms of gastrointestinal infection, CDI needs to be alerted and treated aggressively to improve the prognosis.

## Data Availability

The raw data supporting the conclusions of this article will be made available by the authors, without undue reservation.
